# Mutations in histone modulators are associated with prolonged survival during azacitidine therapy

**DOI:** 10.18632/oncotarget.7899

**Published:** 2016-03-03

**Authors:** Magnus Tobiasson, Donal P. McLornan, Mohsen Karimi, Marios Dimitriou, Monika Jansson, Asmaa Ben Azenkoud, Martin Jädersten, Greger Lindberg, Hani Abdulkadir, Austin Kulasekararaj, Johanna Ungerstedt, Andreas Lennartsson, Karl Ekwall, Ghulam J. Mufti, Eva Hellström-Lindberg

**Affiliations:** ^1^ Center for Hematology and Regenerative Medicine, Department of Medicine, Karolinska Institutet, Karolinska University Hospital Huddinge, Huddinge, Sweden; ^2^ Department of Haematological Medicine, King's College Hospital NHS Foundation Trust, London, United Kingdom; ^3^ Department of Haematological Medicine, King's College, London, United Kingdom; ^4^ Department of Medicine, Karolinska Institutet, Karolinska University Hospital Huddinge, Huddinge, Sweden; ^5^ Department of Biosciences and Nutrition, Karolinska Institutet, Stockholm, Sweden

**Keywords:** myelodysplastic syndrome, azacitidine, hypomethylating therapy, next-generation sequencing, molecular marker

## Abstract

Early therapeutic decision-making is crucial in patients with higher-risk MDS. We evaluated the impact of clinical parameters and mutational profiles in 134 consecutive patients treated with azacitidine using a combined cohort from Karolinska University Hospital (n=89) and from King's College Hospital, London (n=45). While neither clinical parameters nor mutations had a significant impact on response rate, both karyotype and mutational profile were strongly associated with survival from the start of treatment. IPSS high-risk cytogenetics negatively impacted overall survival (median 20 vs 10 months; p<0.001), whereas mutations in histone modulators (*ASXL1, EZH2*) were associated with prolonged survival (22 vs 12 months, p=0.01). This positive association was present in both cohorts and remained highly significant in the multivariate cox model. Importantly, patients with mutations in histone modulators lacking high-risk cytogenetics showed a survival of 29 months compared to only 10 months in patients with the opposite pattern. While *TP53* was negatively associated with survival, neither *RUNX1*-mutations nor the number of mutations appeared to influence survival in this cohort. We propose a model combining histone modulator mutational screening with cytogenetics in the clinical decision-making process for higher-risk MDS patients eligible for treatment with azacitidine.

## INTRODUCTION

In Europe, azacitidine is approved for the treatment of patients with higher-risk myelodysplastic syndromes (MDS), where improvements in survival have been documented [1, 2]. The drug is an inhibitor of DNA methyl transferase resulting in reduced DNA methylation but, although believed to be its principal mode of action, other effects including immunomodulation have been identified and the exact mechanisms of action of azacitidine remain unknown [3-7]. Despite this therapeutic advance, the median survival for higher-risk MDS patients remains short, approximately 12 months, as demonstrated by the Swedish population-based registry for intermediate-2 and high-risk MDS (http://www.cancercentrum.se/inca).

The genetic landscape of the MDS-genome has been well characterized in recent years and some 40 recurrently mutated genes including epigenetic regulators, splicing factors, cohesion factors, transcription factors and others have been identified as driver mutations [8-11]. Several of these e.g. *ASXL1, EZH2, RUNX1* and *TP53* are associated with significantly shorter survival in several MDS cohorts [8-10, 12-14].

Around fifty percent of patients with higher-risk MDS, and AML with dysplastic features and 20-29% marrow blasts, respond to azacitidine as defined by the International Working Group (IWG) criteria [2, 15-17]. Basic clinical data such as morphology and cytogenetics give sparse predictive information, although blast count >15%, extensive transfusion requirements, abnormal karyotype and previous therapy with cytarabine have been reported as negative predictors of response [15, 18, 19]. Although several studies report higher response rates for *TET2*–mutated patients, the presence of this mutation has not been associated with prolonged survival [19-21]. Mutated *ASXL1*, reported as a negative survival factor in numerous studies of heterogeneous MDS cohorts undergoing unspecified therapies [8-10, 14], was associated with shorter survival in a study by Traina et al, evaluating an azacitidine treated cohort (n=92, of which around 40 were higher-risk MDS), but had no impact on survival in a larger cohort undergoing hypomethylating agent (HMA) therapy (n=213, of which 113 were higher-risk MDS) [19, 20]. Of note, both these studies included a significant number of patients with lower-risk MDS and several patients were treated with decitabine, either sequentially or instead of azacitidine.

Within this study, we evaluated the impact of clinical and mutational parameters on response and survival in a large cohort of patients with comprehensive long-term follow-up receiving azacitidine as first-line treatment according to the European label and guidelines (Malcovati et al, Blood 2013). We report for the first time that mutations involved in genes encoding for histone modulating enzymes are associated with a significantly improved survival for patients undergoing azacitdine therapy and, together with cytogenetic analysis, provide a simple model to aid clinical decision-making.

## RESULTS

### Baseline characteristics and response

We included 134 consecutive patients from a mixed population of patients with an indication for azacitidine as first-line treatment; see Table 1 for patient characteristics. According to IPSS, the majority of patients had intermediate-2 risk (n=68) or high risk (n=38) disease. A total of 18 patients were categorized as intermediate-1 risk but were considered eligible for azacitidine either because of elevated blast count, high-risk cytogenetics or rapid deterioration of cytopenia according to European guidelines [27]. Using IPSS-R, 4, 11, 30 and 79 patients belonged to the low, intermediate, high or very high risk groups respectively. Ten patients could not be categorized according to IPSS as they were classified as CMML with white blood cell count (WBC) > 12×10^9^/L; of these, eight were CMML-II and two CMML-I. Responses were evaluated according to IWG criteria as CR (n=30), mCR (n=17), PR (n=8), HI (n=20), SD (n=23) and PD (n=24). Patients with CR, mCR, PR and HI were considered as responders while the remaining patients were considered non-responders. Twelve patients (11%) were not evaluated for response due to early death. All patients had received ≥1 dose of azacitidine. Median number of cycles administered was 7 (range 1-45). Patient characteristics according to hospital (Karolinska or King's College London cohorts) are displayed in Table S2.

**Table 1:** Patient characteristics

Age at start of Azacitidine	70.5 (35-88)
Disease duration (months), median (range)	4 (0-179)
Therapy related, n	17
Transfusion dependent, n	82
WHO subgroups	
RA	1
RCMD +/− RS	16
RAEB-I	27
RAEB-II	60
MDS-AML, ≤ 30% blasts	8
AML with multilinear dysplasia, ≤ 30% blasts	7
CMML type 1	3
CMML type 2	9
MDS/MPN	2
MDS-U	1
Marrow blast %, median (range)	11 (0-30)
Cellularity %, median (range)	70 (10-100)
ANC, median (range)	1.5×10^9^/L (0-30.5)
Plt, median (range)	69×10^9^/L (5-1237)
IPSS cytogenetic risk group	
Favorable, n	59
Intermediate, n	20
Adverse, n	55
IPSS risk group	
Low, n	0
Intermediate-I, n	18
Intermediate-II, n	68
High, n	38
IPSS-R risk group	
Low	4
Intermediate	11
High	30
Very high	79
Number of cycles given, median (range)	7 (1-45)
Response	
Complete remission, n	30
Marrow complete remission, n	17
Partial remission, n	8
Hematological improvement, n	20
Stable disease, n	23
Progression, n	24
Not evaluated, n	12

Abbreviations: WHO, world health organization; RA, refractory anemia; RARS, Refractory anemia with ringed sideroblasts; RCMD, refractory cytopenia with multilineage dysplasia; RCMD-RS, RCMD with ringed sideroblasts; RAEB-I, Refractory anemia with excess of blasts; MPN, myeloproliferative neoplasm; CMML chronic myelomonocytic leukemia; CML chronic myeloid leukemia; AML Acute myeloid leukemia; IPSS, international prognostic score system.

Of the total cohort (n=134), 110 (82%) had ≥ 1 mutation of which the most common were *ASXL1* (n=29), *TET2* (n=26), *SRSF2* (n=24), and *TP53* (n=20). Mutational frequencies of the most frequent mutations did not differ significantly between the two cohorts (see table S4A and S4B). According to IPSS cytogenetic profiling, 59, 20 and 55 patients had low-risk, intermediate-risk and high-risk cytogenetics, respectively. Eleven patients had neither mutations, nor cytogenetic abnormalities.

### Weak association between routine clinical parameters / mutational profile and response to azacitidine treatment

We observed a trend towards shorter pre-treatment disease duration among responders compared to non-responders (median 3 vs 7 months, p=0.055). No other clinical parameters, including morphological or cytogenetic characteristics were associated with azacitidine response rates. Interestingly, known unfavorable prognostic markers including adverse cytogenetics, or therapy-related disease were not significantly associated with response to azacitidine in this cohort (see Table 2).

**Table 2:** Pre-treatment variables associated with response

Variable		Response	No response	p-value
Age, median (range)		72 (35-88)	69 (50-85)	0.40
Disease duration, median (range)		3 (0-117)	7 (0-179)	0.06
Marrow blasts %, median (range)		11 (0-30)	12 (1-25)	0.66
Cellularity %, median (range)		70 (10-100)	70 (10-100)	0.41
Absolute neutrophil count, ×10^9^/L, median (range)		1.3×10^9^/L (0.1-15.8)	1.9×10^9^/L (0-30.5)	0.39
Platelets, ×10^9^/L, median (range)		70×10^9^/L (5-1237)	65×10^9^/L (5-790)	0.87
Transfusion dependent, n (%)	Yes	42 (56)	40 (68)	0.23
	No	33 (44)	19 (32)	
Therapy-related, n (%)	Yes	10 (13)	7 (12)	1.00
	No	65 (87)	52 (88)	
IPSS cytogenetic risk group, n (%)	Favorable	37 (49)	22 (37)	0.38
	Intermediate	10 (13)	10 (17)	
	Adverse	28 (37)	27 (46)	
IPSS risk score, n (%)	Low	0 (0)	0 (0)	0.55
	Int-1	11 (16)	7 (12)	
	Int-2	38 (57)	30 (53)	
	High	18 (26)	20 (35)	
Number of mutations, median (range)		2 (0-5)	1 (0-5)	0.98
*Mutations, n (%)*				
*ASXL1*	Yes	19 (25)	10 (17)	0.34
	No	56 (75)	49 (83)	
*TET2*	Yes	18 (24)	8 (14)	0.19
	No	57 (76)	51 (86)	
*SF3B1*	Yes	4 (5)	5 (8)	0.71
	No	71 (95)	54 (92)	
*SRSF2*	Yes	13 (17)	11 (19)	1.00
	No	62 (83)	48 (81)	
*IDH1/2*	Yes	9 (12)	8 (14)	0.99
	No	66 (88)	51 (86)	
Epigenetic factor mutations	Yes	47 (63)	29 (49)	0.16
*(TET2, DNMT3A, IDH1/2, MLL, EZH2, ASXL1)*	No	28 (37)	30 (51)	
Histone modulator mutations	Yes	25 (33)	11 (19)	0.09
*(ASXL1, EZH2)*	No	50 (67)	48 (81)	
DNA methylation mutations	Yes	30 (40)	19 (32)	0.45
*(TET2, DNMT3A, IDH1/2)*	No	45 (60)	40 (68)	
Splicing factor mutations	Yes	21 (28)	22 (37)	0.34
*(SF3B1, SRSF2, PRPF40B, U2AF1, U2AF35, ZRSR2)*	No	54 (72)	37 (63)	
Cohesion factor mutations	Yes	2 (3)	2 (3)	1.00
*(STAG2, SMC3, PDS5B)*	No	73 (97)	57 (97)	
Signaling factor mutations	Yes	10 (13)	15 (25)	0.12
*(JAK2, MPL, CBL, FLT3, NRAS, WT1, SH2B3)*	No	65 (87)	44 (75)	
Transcription factor mutations	Yes	14 (19)	8 (14)	0.58
*(RUNX1, ETV6, CEBPA, BCOR)*	No	61 (81)	51 (86)	

Abbreviations: IPSS, International prognostic score system; Int, Intermediate.

Furthermore, no single mutation or group of mutations was significantly associated with response (i.e. any of CR, mCR, PR or HI). *TET2*-mutated patients have in previous publications been reported to have higher response rates [20, 21]. In our cohort, although the response rate was higher for these patients, the difference was not significant. (69% vs. 53%, p=0.19).

We next grouped genes into epigenetic factors, which in turn were further divided into DNA methylation factors and histone modulators, splicing factors, transcription factors, signaling factors and cohesion factors (see Table 2 for genes included in each group). Patients with epigenetic mutations had a weak trend for higher response rates (62% vs 48%, p=0.16). When the group was divided into patients with DNA methylation mutations and histone modulator mutations, respectively, a trend for higher response rates was present for those with histone modulator mutations (69% vs 51%, p=0.09), but not for DNA methylation factors (61% vs 47%, p=0.45). The number of mutations did not differ between responders and non-responders (median 2 (0-5) vs 2 (0-5); p=0.98). Due to the weak association between tested variables and response rates, multivariate analysis was not performed. Table 2 summarizes the univariate analyses with response as endpoint and Table S3 displays data as dichotomized by institution.

### Factors associated with survival in the univariate analysis

Estimated median overall survival for the whole cohort was 17 months (95% confidence interval (CI): 14-20 months), which is shorter than in the AZA001 study (19-20 months) but longer than in the French GFM cohort (13.5 months) [2, 15]. Median follow up time for all patients was 14 months, and median follow-up for surviving patients was 23 months. Twenty-three patients underwent allogeneic stem cell transplantation (SCT) after a median of 8 months (range 2-45) from start of azacitidine treatment; when censoring for SCT the estimated median survival in the whole cohort was 14 months (95% CI: 11-17 months). In the subsequent analyses we used data censored for transplantation. Patients who did not achieve a response (i.e. CR, mCR, PR or HI) had, as expected, a significantly shorter survival compared to responders (median 10 months vs. 20 months, p=<0.001).

Among clinical pre-treatment variables, disease duration was associated with survival when used as a continuous variable in a univariate cox model (p=0.04; HR 1.01 (95% CI 1.00-1.02)). However, when dichotomizing this variable into above or below median, it lost statistical significance in the log-rank test (14 vs 17 months; p=0.44). Patients with thrombocytopenia (platelets <60×10^9^/L; median value in the entire cohort), showed a non-significant trend towards shorter survival (12 vs 17 months; p=0.067). Patients belonging to IPSS-R cytogenetics risk groups ‘high’ or ‘very high’ had a significantly shorter survival compared to the very good, good or intermediate risk groups (10 vs 20 months; p<0.001).

The presence of mutations in any of the epigenetic modulators was associated with improved survival (19 vs 12 months; p=0.03). After dividing mutations into those affecting DNA methylation versus histone modulators, the former group did not show a significant impact on survival (14 vs 14 months; p=0.64). By contrast, patients with mutations in histone modulators (*ASXL1 or EZH2*), showed a significantly longer survival (22 vs 12 months; p=0.01). This difference remained significant for patients with *ASXL1* mutations (n=29; survival 29 vs 12 months; p=0.026) while patients with mutations in EZH2 (n=12) showed a trend towards longer survival (20 vs 14 months; p=0.37). When separating the patient material into higher-risk disease (IPSS Int-2, IPSS-high, CMML-2 and AML with multilineage dysplasia; n=114) and lower-risk disease (IPSS Int-1 and CMML-1; n=20), histone modulator mutations had a strong impact on survival in the higher-risk cohort (20 vs 12 months; p=0.02). They were also associated, although not significantly, with longer survival in the lower-risk cohort (32 vs 17 months; p=0.47; n=20). It should be noted that the MLL-gene, which also possesses histone modulating activity, was only assessed in the Karolinska cohort (n=2) and was therefore not included in the histone modulator group analysis. As expected, TP53 had a significant negative impact on survival (9 vs 17 months; p<0.001) but, interestingly, neither *RUNX1* mutations nor the number of mutations, previously described poor-prognostic findings, were associated with shorter survival [8-10].

**Figure 1:**
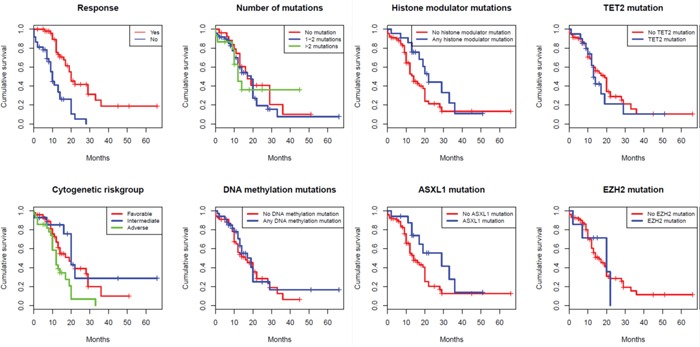
Survival curves using Kaplan-Meier estimation

**Figure 2:**
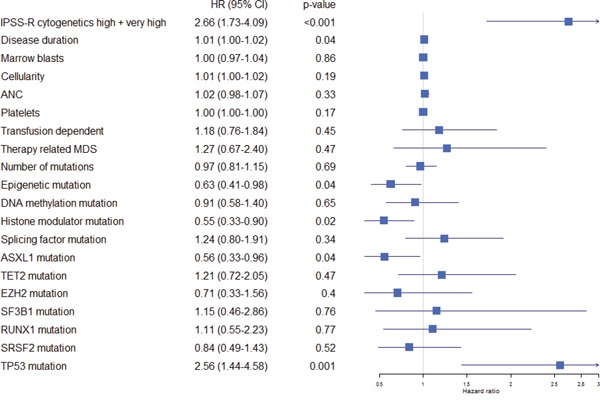
Forest plot indicating hazard ratio including confidence interval for all pre-treatment variables The hazard ratios were retrieved using cox univariate regression models for each variable analyzed separately. Abbreviations: IPSS International prognostic score system, ANC absolute neutrophil count, HR hazard ratio, CI confidence interval.

The negative impact on survival of adverse cytogenetics (IPSS-R high or very high; 10 vs 23 months; p<0.001), and bone marrow cellularity (<70 or ≥70%; 14 vs 31; p=0.01) also remained significant when the patients were not censored at the time of SCT. Interestingly, the strength of the statistical association of histone modulator mutations then became less pronounced (29 vs 14 months; p=0.077), supporting the notion that mutations in histone modulators may have a specific role in the response to azacitidine. See Table 3 and Figure 2 for a list of variables analyzed using survival as an endpoint, Table S5 for the Karolinska and King's College cohort separated, and Figure 1 and Figure S1 for survival plots respectively. The survival curve for histone modulators mutations without censoring for SCT is presented in Figure S2.

**Table 3:** Variables associated with survival

	Estimated median survival (months)	Univariate p-value	Cox regression p-value	Hazard ratio (95% CI)
Response: Yes vs No	20 vs 10	<0.001		
IPSS-R cytogenetic risk group: VG + Good+Int vs High + VH	20 vs 10	<0.001	<0.001*	3.46 (2.09-5.59)
WHO group: MDS vs MDS/MPD vs AML with multilinear dysplasia	14 vs 20 vs 28	0.61		
Disease duration ≥ 4 months: Yes vs No	14 vs 17	0.44	0.003**	1.01 (1.00-1.02)**
Marrow blasts ≥ 11%: Yes vs No	14 vs 14	0.7		
Cellularity ≥ 70%: Yes vs No	14 vs 20	0.2	0.05**	1.01 (1.00-1.02)**
ANC ≥ 1.3: Yes vs No	14 vs 17	0.32		
Platelets ≥ 60: Yes vs No	17 vs 12	0.07		
Transfusion dependent: Yes vs No	13 vs 17	0.43	0.04	1.70 (1.03-2.80)
Therapy related: Yes vs No	17 vs 14	0.44		
Number of mutations: 0 vs 1 vs ≥ 2	17 vs 12 vs 17	0.64		
Epigenetic mutation: Yes vs No	19 vs 12	0.03		
DNA methylation mutation: Yes vs No	14 vs 14	0.64		
Histone modulator mutation: Yes vs No	22 vs 12	0.01	0.01	0.50 (0.30-0.85)
Splicing factor mutation: Yes vs No	13 vs 17	0.31	0.05	1.63 (1.011-2.63)
Transcription factor mutation: Yes vs No	16 vs 14	0.93		
Signaling factor mutation: Yes vs No	19 vs 14	0.60		
Cohesin factor mutation: Yes vs No	19 vs 14	0.20		
*ASXL1* mutation: Yes vs No	29 vs 12	0.03		
*TET2* mutation: Yes vs No	13 vs 16	0.45		
*EZH2* mutation: Yes vs No	20 vs 14	0.37		
*SF3B1* mutation: Yes vs No	13 vs 16	0.35		
*RUNX1* mutation: Yes vs No	17 vs 14	0.76		
*SRSF2* mutation: Yes vs No	20 vs 14	0.5		
*TP53* mutation: Yes vs No	9 vs 17	<0.001		

Univariate analyses used the log-rank test.

* Comparing the combined IPSS-R cytogenetic risk groups high and very high vs all other groups.

** Disease duration, marrow blasts, cellularity, ANC and TPK were analyzed as a continuous variable in the cox model.

Abbreviations: CI, confidence interval; CR, complete remission; mCR, marrow complete remission; PR, partial remission; HI, hematological improvement; SD, stable disease; PD, progressive disease; IPSS International prognostic score system; ANC, absolute neutrophil count; MPD myeloproliferative disease; VG very good; VH very high.

### High-risk cytogenetics and mutations in chromatin modifiers remain independent predictors for survival

In a cox model including all parameters as defined in the methods section, adverse cytogenetics (IPSS-R high or very high; p<0.001; HR 3.46 (2.09-5.59 95% CI)) and histone modulator mutations (p=0.01; HR 0.50 (0.30-0.85 95% CI)) remained strong predictors of survival. Other variables associated with survival were: cellularity (p=0.05; HR 1.01; 95% CI 1.00-1.02), disease duration (p=0.003; HR 1.01 (1.00-1.02)), transfusion dependency (p=0.04; HR 1.70 (1.03-2.80 95% CI)), splicing factor mutation (p=0.05; HR 1.63 (1.01-2.63) 95% CI). Neither transfusion dependency nor splicing factor mutation displayed an association with survival on univariate analyses (p=0.43 and p=0.31, respectively) and the impact of these parameters on survival is unclear. As expected, *TP53* showed a strong association with survival in the univariate analysis but not in the multivariate analyses, probably due to co-linearity with high-risk cytogenetics.

**Figure 3:**
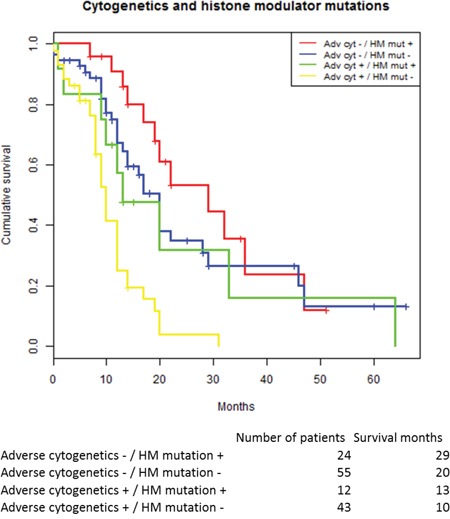
Kaplan-Meier estimated survival stratified for the two dominant predictors in the cox regression model: Adverse cytogenetics and histone modulator mutations Abbreviations: HM histone modulator mutation.

We used the two strongest predictors, adverse cytogenetics and histone modulator mutations, to allocate patients to four prognostic groups: dependent on cytogenetic status (IPSS-R high or very high) and presence of mutations in histone modulators (HM). Survival in the four risk groups were: cyt+/HM+ 29 months; cyt-/HM- 20 months; cyt+/HM+ 13 and cyt+/HM- 10 months, respectively, see Figure 3. The differences in survival between the groups were highly significant (p<0.001). The cumulative response rates to azacitidine for patients within the prognostic groups were 73% (19 out of 26) for the best prognostic group (histone modulator mutation but no adverse cytogenetics), 53% (28 out of 53) and 58% (7 out of 12) for the two intermediate prognostic groups; and 49% (21 out of 43) for the least favorable prognostic group.

## DISCUSSION

Patients with higher-risk MDS have an overall poor outcome despite the survival benefits achieved by azacitidine therapy. The median survival in unselected patient cohorts is only around one year, hence the major challenge for treating physicians remains how to prolong survival [2, 15, 28]. Around 50% of patients with higher-risk MDS respond to azacitidine, but patients who are HMA-refractory have an expected survival of only 6 months, [29]. Accessible clinical tools that can therapeutically stratify patients upfront therefore remain an urgent unmet need. Since patients without response but with stable disease during azacitidine treatment demonstrate survival benefits as demonstrated by a post-hoc analysis of the AZA-001-study, we chose survival from start of treatment as the most appropriate endpoint for this study assessing the benefits of azacitidine treatment [30].

The aim was to identify clinically relevant biomarkers to determine the efficacy of azacitidine. This study uses a combined cohort of azacitidine-treated patients from the Karolinska University Hospital (n=89) and King's College Hospital (n=45). The vast majority of these patients fell within the recommended European label for the drug and had higher-risk MDS or AML with 20-30% blasts and dysplastic features. Importantly, and in contrast to other published studies, all patients received azacitidine as first-line treatment with the aim to give a minimum of 6 cycles before response evaluation. We believe that this approach, and the fact that patients were assessed according to an intention-to-treat basis, makes our cohort more homogeneous and similar to the AZA-001 cohort than other published retrospective studies. Moreover, the fact that two different sequencing platforms rendered similar results strengthens the possibility to extrapolate these data to broader clinical practice.

Interestingly, no single variable was significantly associated with response, as defined by the IWG criteria, although patients with longer disease duration prior to azacitidine, and transfusion dependency displayed a trend towards worse response rates. In line with previous studies, patients with *TET2* mutations displayed somewhat better response rates [19-21].

For the subsequent analyses, we used survival as the primary endpoint and, unsurprisingly, saw a poorer outcome in patients with adverse cytogenetics and *TP53* mutations, both well-known adverse prognostic factors in MDS. The novel finding was, however, the strong positive impact of histone modulator mutations on survival (22 vs 12 months, p=0.01). This association was present both in the combined cohort of Karolinska and King's, as well as in each of the cohorts analyzed separately and, importantly, remained independent from the negative impact of high-risk cytogenetics. Patients with two negative biomarkers showed a median survival of only 10 months, potentially making them eligible for consideration of alternative therapeutic agents.

*ASXL1* was the most frequent mutation within the histone modulator group and also showed a significant impact on survival (p=0.03). The pivotal finding of this study is that a molecular marker, which repeatedly has been associated with poor survival, appears as a positive biomarker in both the primary and validation cohorts of azacitidine-treated higher-risk MDS patients. The lack of a negative effect of *RUNX1* mutations and total number of mutations also indicates that azacitidine may have specific biological effects on the MDS tumor clone [8]. By contrast, mutations in modulators of DNA methylation showed no association with survival following azacitidine treatment.

These results contrast findings in previous studies investigating the effects of *ASXL1* mutations on survival in azacitdine-treated patients [19, 20]. An important difference is the higher proportion of lower-risk patients, around 50%, in those studies compared to only 15% in the present study with the Intermediate-1 risk patients included in our study being treated according to the European guidelines because of significant additional risk factors [27]. Moreover, in contrast to previous studies, all patients in our cohort received first-line treatment Azacitidine without previous or subsequent hypomethylating therapies. They well treated with a median of 7 cycles of azacitidine given in line with guidelines [27]. Survival was analyzed using an intention-to treat perspective in order to avoid selection bias. Since there are conflicting results of the impact of *ASXL1* on survival, larger studies are certainly warranted. Interestingly, two recent publications indicate a better prognosis for *ASXL1*-mutated patients after SCT (however without significance calculation in the study by Fu *et al* and not statistically significant in the study by Bejar *et al*) supporting that *ASXL1* mutations, despite being a negative prognostic factor in mixed MDS cases, can be a positive factor for survival during treatment [31, 32].

Our data suggests that patients carrying mutations in histone modulators are more sensitive to the effects of azacitidine. Interestingly, we have previously shown that azacitidine exerts changes on chromatin structure and since histone modulator mutations are associated with increased sensitivity to azacitidine, it will be important to further delineate the chromatin structure in MDS subtypes and how this is affected by azacitidine [3]. Whether decitabine, used sequentially or instead of azacitidine in the other studies, has a differential effect cannot be excluded [19, 20]. Very recent data indicates that *ASXL1* mutation results in lower expression of the tumor suppressor gene *p15INK4b* which has been shown to be hypermethylated in MDS [33, 34]. We have previously analyzed DNA methylation (Illumina 450k methylation array) in primary MDS cells cultured with azacitidine and observe a decrease in DNA methylation in all probes annotated to *p15INK4b* (mean reduction in β-value = 0.023) [35]. A hypothesis that may be tested is that reduction of DNA methylation in *ASXL1^mut^* cells induces a greater increase in expression of *p15INK4b* compared to *ASXL1^wt^* cells.

With the advent of next-generation sequencing, new potential biomarkers are rapidly being incorporated into routine clinical practice and are likely to influence patient management and therapeutic decisions. Adverse cytogenetics remains a well-established prognostic marker with documented negative effects on the outcome of both conventional chemotherapy and azacitidine [36, 37]. We hereby report that histone modulator mutations are associated with significantly prolonged survival following azacitidine treatment and, importantly, that this effect is independent from that of high-risk cytogenetics. The combination of these two variables, easily detected by routine karyotyping and limited targeted sequencing, respectively, provide a valuable model that, if confirmed in independent patient cohorts, can be used in routine clinical practice to guide therapeutic decision-making in patients eligible for azacitidine. In particular, patients with high-risk cytogenetics lacking mutations in histone modulators may be evaluated for alternative therapeutic pathways.

## MATERIALS AND METHODS

### Patient population

The study population included 134 patients, diagnosed with either MDS, AML secondary to MDS or primary AML with multilineage dysplasia and 20-30% blasts, treated with azacitidine. Consecutive patients were recruited retrospectively from two large centers: one cohort from Karolinska University Hospital, Stockholm, Sweden (n=89) and a second cohort from King's College Hospital, London, United Kingdom (n=45). In order to avoid selection bias and to adopt an “intention-to-treat” approach to the analysis, patients were considered evaluable if they had received ≥1 dose (day) of azacitidine. Treatment scheduling was conducted according to the European label, aiming for a minimum of 6 cycles before response evaluation. Clinical parameters evaluated included age, disease duration, peripheral blood counts, transfusion dependency, therapy related disease, bone marrow blasts, marrow cellularity, International Prognostic Scoring System (IPSS) risk group, IPSS-Revised (IPSS-R) and IPSS cytogenetic risk group determined by metaphase cytogenetics. Morphological and cytogenetic assessments were made using local, validated laboratory procedures and all patients underwent consensus diagnosis at a multidisciplinary conference. Response to azacitidine was evaluated using IWG criteria and patients were categorized as either: complete remission (CR), marrow complete remission (mCR), partial remission (PR), hematological improvement (HI), stable disease (SD) or progressive disease (PD). Responders were defined as achieving at least HI and responses were defined as the best response during the whole course of treatment. The Ethical committees at the Karolinska Institute and King's College Hospital, respectively, approved the study. All patients provided fully informed consent.

### Mutational analysis

#### The Karolinska cohort

Patients were analyzed for 42 genes recurrently mutated in myeloid disorders using Haloplex^TM^ target enrichment technology (Agilent Technologies, CA, United States) followed by high throughput sequencing, see table S1 for a list of included genes. Briefly, mononuclear cells (MNCs) were isolated from bone marrow aspirates by Lymphoprep^®^ density gradient centrifugation. Genomic DNA was extracted from 1×10^6^ CD34- cells or MNCs using Gene Elute genomic DNA extraction kit (Sigma-Aldrich, Stockholm, Sweden). Haloplex^TM^ target enrichment kit G9901A/B was designed using SureDesign^tm^ wizard available by Agilent (https://earray.chem.agilent.com/suredesign/) and we achieved 99.2% coverage of the 42 selected genes. All samples were individually barcoded during enrichment and sequenced using Illumina HiSeQ 2000 system at the Sci-Life lab, Stockholm, Sweden. Sequencing reads were mapped over Human genome 19 by Bowtie and the variants were called using SAMTOOLS [22, 23]. The minimum of variant reads to consider was 20 with a minimum allelic burden of 5%. Sequence variations were annotated and functionally classified using ANNOVAR [24]. Variants previously reported as germline polymorphisms in the 1000 genome and the ESP5400 databases were excluded [25, 26]. Finally, variants located in none coding regions as well as synonymous variants were filtered out. Variant allele ratio was calculated for each mutation identified as number of variant reads divided by total reads.

#### The King's College London cohort

DNA was extracted from bone marrow CD34+ cells or MNCs using the QIAamp DNA extraction kit (Qiagen, Limburg, Netherlands) according to the manufacturer's protocol. A targeted ‘24 gene’ myeloid gene panel was used for the analysis of presentation samples from MDS and AML patients (n=45). Target enrichment was achieved using an in-house True SeqCustom Amplicon (TSCA) design (Illumina, San Diego, USA). The targeted region consisted of a total of 71Kb represented by 295 amplicons. Pooled library targets were sequenced in batches of 24 samples on the MiSeq Instrument using version 3.0 MiSeq sequencing reagents (Illumina, San Diego, USA). Minimum read depth threshold was 150 reads; lower limit of sensitivity was 5-10% variant allele frequency. All variants of unknown significance were excluded. Genes in this panel included: *ASXL1* exons 1-12, *CBL* exons 7-9, *CEBPA* all coding exons, *DNMT3A* all coding exons, *ETV6/TEL* all coding exons, *EZH2* all coding exons, *FLT3* exons 14+20, *GATA2* all coding exons, *IDH1* exon 4, *IDH2* exon 4, *JAK2* exons 12+14, *KDM6A* all coding exons, *KIT* exons 17, *KRAS* exons 2+3, *NPM1* exon 12, *NRAS* exons 2+3, *RUNX1* all exons except 1+2, *SF3B1* exons 12 to 16, *SRSF2* exon 1, *STAG2* all coding exons, *TET2* all coding exons, *TP53* all coding exons, *U2AF1* exons 2+6 and *ZRSR2* all coding exons.

### Statistics

Continuous variables were expressed using the mean ± Standard Deviation (SD) or the median (range) depending on whether distributions were normal or not. Frequency tables were used for summarizing categorical variables. Statistical methods used for association studies were the t-test for normally distributed data, Mann-Whitney U-test for non-normally distributed data and a Chi-squared or Fisher's exact test for categorical data. Time-to-event data were analyzed using the Kaplan–Meier method. Overall survival (OS) was defined from the time of start of treatment to the date of death or when last seen. Patients were censored at the time of allogeneic stem cell transplantation. A Cox proportional hazard model was used to assess the effect of multiple factors on survival where a backward elimination algorithm was used to identify independent predictors. Included parameters in the Cox model were: IPSS adverse cytogenetic risk group, WHO group (MDS vs MDS/MPD vs AML with multilineage dysplasia), disease duration, marrow blast percentage, marrow cellularity, ANC, platelet count, transfusion dependency, therapy related MDS, number of mutations, epigenetic mutations, DNA methylation mutations, histone modulator mutations, splice factor mutations, *RUNX1* mutation and *TP53* mutation. Data on marrow cellularity was missing in 8 cases. For these cases, the median value was used in the multivariate analysis.

All statistical calculations were performed using R version 3.1.1 and SPSS version 22.0 (IBM, NY, United States).

## SUPPLEMENTARY TABLES AND FIGURES






