# Supplementary material for: Mutations in histone modulators are associated with prolonged survival during azacitidine therapy
Source: Oncotarget. 2016 Mar 3;7(16):22103–15. doi: 10.18632/oncotarget.7899 (PMC5008347; doi:10.18632/oncotarget.7899)
Supplement: Supplementary file 1 [file oncotarget-07-22103-s001.pdf]

SUPPLEMENTARY TABLES AND FIGURES

Supplementary Table S1A: Mutations included for sequencing, Karolinska cohort

ASXL1	GATA1	MPL	SH2B3	WT1
BCOR	GATA2	NPM1	SMC1A	ZRSR2
CBL	GATA3	NRAS	SMC3	
CEBPA	IDH1	PDS5B	SRSF2	
CSF3R	IDH2	PRPF40B	STAG1	
DNMT3A	JAK2	RAD21	STAG2	
EPOR	KDM6A	RUNX1	TET2	
ETV6	KIT	SF1	TP53	
EZH2	KRAS	SF3A1	U2AF1	
FLT3	MLL	SF3B1	U2AF2	

Supplementary Table S1B: Mutations included for sequencing, King's cohort

ASXL1	FLT3	KIT	SRSF2
CBL	GATA2	KRAS	STAG2
CEBPA	IDH1	NPM1	TET2
DNMT3A	IDH2	NRAS	TP53
ETV6/TEL	JAK2	RUNX1	U2AF1
EZH2	KDM6A	SF3B1	ZRSR2

Supplementary Table S2: Mutational frequency (224 mutations identified in total)

ASXL1	29	PRPF40B	3
BCOR	4	RUNX1	17
CBL	5	SF3A1	1
CEBPA	2	SF3B1	9
DNMT3A	10	SH2B3	2
ETV6	2	SMC3	2
EZH2	12	SRSF2	24
FLT3	2	STAG2	2
IDH1	10	TET2	26
IDH2	7	TP53	20
JAK2	8	U2AF1	5
KRAS	3	U2AF35	4
MLL	2	WT1	1
MPL	1	ZRSR2	1
NRAS	9	no mutation	24
PDS5B	1		

Supplementary Table S3A: Patient characteristics, KI's cohort

Age at start of Azacitidine	73 (35-88)
Disease duration (months), median (range)	2 (0-179)
Therapy related, n	12
Transfusion dependent, n	54
WHO subgroups	
RA	1
RCMD +/- RS	8
RAEB-I	16
RAEB-II	38
MDS-AML	7
AML with multilinear dysplasia	7
CMML type 1	3
CMML type 2	7
MDS/MPN	2
MDS-U	0
Marrow blast percentage, median (range)	12 (0-30)
Cell percentage, median (range)	70 (10-100)
ANC, median (range)	1.5 (0.1-30.5)
Plt, median (range)	67 (5-1237)
IPSS cytogenetic risk group	
Favorable, n	47
Intermediate, n	14
Adverse, n	28
IPSS risk group	
Low, n	0
Intermediate-I, n	11
Intermediate-II, n	48
High, n	22
IPSS-R risk group	
Low	0
Intermediate	8
High	24
Very high	49
Number of cycles given, median (range)	8 (1-29)
Response	
Complete remission, n	21
Marrow complete remission, n	12
Partial remission, n	3
Hematological improvement, n	16
Stable disease, n	20
Progression, n	8
Not evaluated, n	9

Supplementary Table S3B: Patient characteristics, King's cohort

Age at start of Azacitidine	68 (45-85)
Disease duration (months), median (range)	7 (1-117)
Therapy related, n	5
Transfusion dependent, n	28
WHO subgroups	
RA	0
RCMD +/- RS	8
RAEB-I	11
RAEB-II	22
MDS-AML	1
AML with multilinear dysplasia	0
CMML type 1	0
CMML type 2	2
MDS/MPN	0
MDS-U	1
Marrow blast percentage, median (range)	10 (0-26)
Cell percentage, median (range)	70 (10-100)
ANC, median (range)	1.5 (0-8.6)
Plt, median (range)	70 (12-241)
IPSS cytogenetic risk group	
Favorable, n	12
Intermediate, n	6
Adverse, n	27
IPSS risk group	
Low, n	0
Intermediate-I, n	7
Intermediate-II, n	20
High, n	16
IPSS-R risk group	
Low	4
Intermediate	3
High	6
Very high	30
Number of cycles given, median (range)	6 (1-45)
Response	
Complete remission, n	9
Marrow complete remission, n	5
Partial remission, n	5
Hematological improvement, n	4
Stable disease, n	3
Progression, n	16
Not evaluated, n	3

Supplementary Table S4A: Pre-treatment variables associated with response, Karolinska cohort.

See Supplementary File S1

Supplementary Table S4B: Pre-treatment variables associated with response, Kings cohort.

See Supplementary File S2

Supplementary Table S5A: Variables associated with survival, Karolinska cohort

Variable	Estimated median survival (months)	Univariate p-value
Response rate: Yes vs No	20 vs 10	<0.001
IPSS cytogenetic risk group: VG + Good+Int vs High + VH	20 vs 12	0.003
Disease duration ≥ 4 months: Yes vs No	13 vs 19	0.16
Marrow blasts ≥ 11%: Yes vs No	17 vs 19	0.88
Cellularity ≥ 70%: Yes vs No	14 vs 20	0.14
ANC ≥ 1.3: Yes vs No	14 vs 28	0.03
Platelets ≥ 60: Yes vs No	19 vs 17	0.19
Transfusion dependent: Yes vs No	14 vs 19	0.7
Therapy related: Yes vs No	17 vs 17	0.46
Number of mutations: 0 vs 1 vs ≥ 2	17 vs 19 vs 12	0.44
Epigenetic mutation: Yes vs No	20 vs 12	0.09
DNA methylation mutation: Yes vs No	19 vs 16	0.67
Histone modulator mutation: Yes vs No	22 vs 14	0.06
Splicing factor mutation: Yes vs No	13 vs 19	0.22
ASXL1 mutation: Yes vs No	29 vs 14	0.07
TET2 mutation: Yes vs No	13 vs 19	0.51
EZH2 mutation: Yes vs No	20 vs 16	0.91
SF3B1 mutation: Yes vs No	13 vs 17	0.6
RUNX1 mutation: Yes vs No	17 vs 17	0.8
SRSF2 mutation: Yes vs No	20 vs 17	0.56
TP53 mutation: Yes vs No	12 vs 17	0.54

Supplementary Table S5B: Variables associated with survival, Kings cohort

Variable	Estimated median survival (months)	Univariate p-value
Response:Yes vs No	20 vs 9	0.002
IPSS cytogenetic risk group: VG + Good+Int vs High + VH	31 vs 9	<0.001
Disease duration ≥ 4 months: Yes vs No	14 vs 12	0.11
Marrow blasts ≥ 11%: Yes vs No	14 vs 12	0.85
Cellularity ≥ 70%: Yes vs No	14 vs 12	0.93
ANC ≥ 1.3: Yes vs No	14 vs 12	0.32
Platelets ≥ 60: Yes vs No	17 vs 12	0.76
Transfusion dependent: Yes vs No	12 vs 17	0.23
Therapy related: Yes vs No	17 vs 12	0.94
Number of mutations: 0 vs 1 vs ≥ 2	14 vs 12 vs 20	0.6
Epigenetic mutation: Yes vs No	14 vs 12	0.19
DNA methylation mutation: Yes vs No	11 vs 12	0.73
Histone modulator mutation: Yes vs No	20 vs 12	0.05
Splicing factor mutation: Yes vs No	12 vs 12	0.93
ASXL1 mutation: Yes vs No	20 vs 12	0.09
TET2 mutation: Yes vs No	9.5 vs 12	0.39
EZH2 mutation: Yes vs No	32 vs 12	0.3
SF3B1 mutation: Yes vs No	NA	NA
RUNX1 mutation: Yes vs No	10 vs 12	0.4
SRSF2 mutation: Yes vs No	14 vs 12	0.59
TP53 mutation: Yes vs No	8 vs 17	<0.001

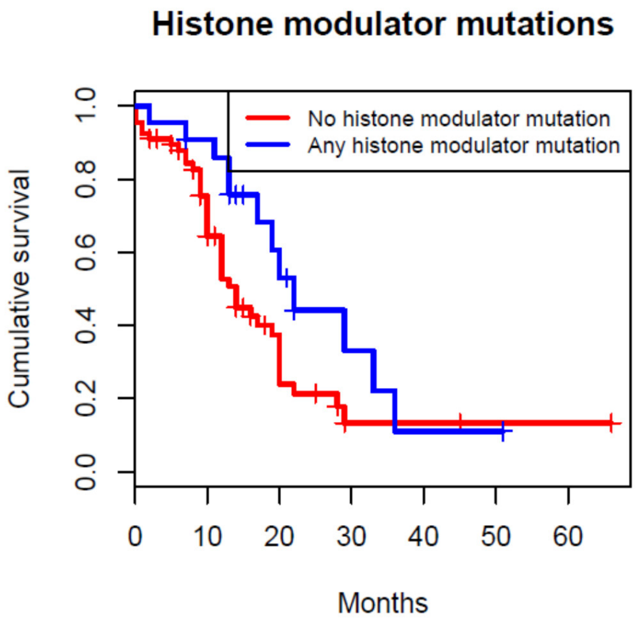

Supplementary Figure S1A: Survival histone modulator mutations, KI's cohort

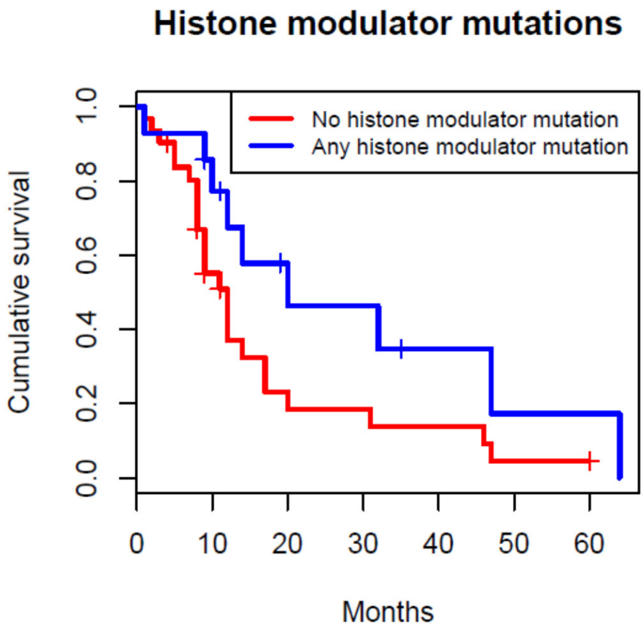

Supplementary Figure S1B: Survival histone modulator mutations, King's cohort

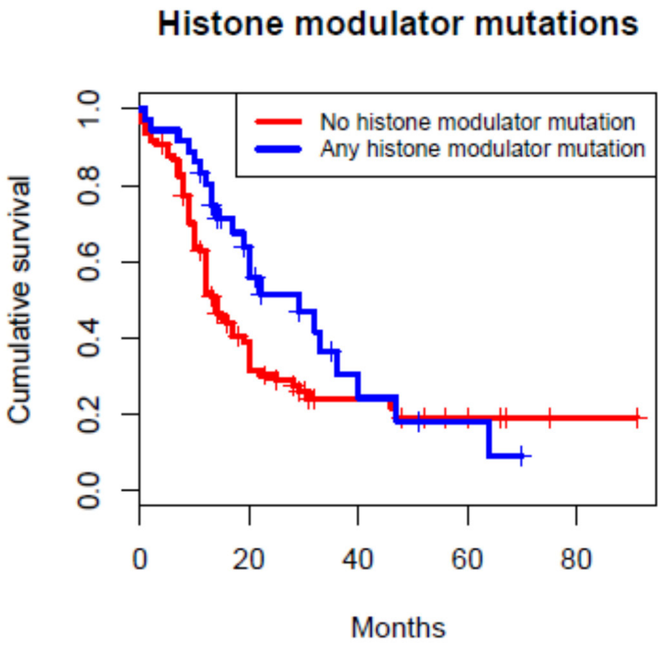

Supplementary Figure S2: Survival histone modulator mutations when patients were not censored for SCT.
