# Supplementary material for: Mutations in histone modulators are associated with prolonged survival during azacitidine therapy
Source: Oncotarget. 2016 Mar 3;7(16):22103–15. doi: 10.18632/oncotarget.7899 (PMC5008347; doi:10.18632/oncotarget.7899)
Supplement: Supplementary file 2 [file oncotarget-07-22103-s002.docx]

| Table S4A: Pre-treatment variables associated with response, Karolinska cohort. | | | |  |
| --- | --- | --- | --- | --- |
|  |  |  |  |  |
| Variable | | Response | No response | p-value |
| Age, median (range) | | 73 (35-88) | 71 (51-85) | 0.67 |
| Disease duration, median (range) | | 2 (0-88) | 5 (0-179) | 0.15 |
| Marrow blasts %, median (range) | | 11 (0-30) | 13 (2-25) | 0.47 |
| Cellularity %, median (range) | | 70 (10-100) | 70 (30-100) | 0.37 |
| Absolute neutrophil count, x10^9^/L, median (range) | | 1.3 (0.1-15.8) | 1.9 (0.1-30.5) | 0.28 |
| Platelets , x10^9^/L, median (range) | | 66 (5-1237) | 88 (5-790) | 0.88 |
| Transfusion dependent, n (%) | Yes | 29 (56) | 25 (68) | 0.37 |
|  | No | 23 (44) | 12 (32) |  |
| Therapy-related, n (%) | Yes | 8 (15) | 4 (11) | 0.76 |
|  | No | 44 (85) | 33 (89) |  |
| IPSS cytogenetic risk group, n (%) | Favorable | 27 (52) | 20 (54) | 0.66 |
|  | Intermediate | 7 (13) | 7 (19) |  |
|  | Adverse | 18 (35) | 10 (27) |  |
| IPSS risk score, n (%) | Low | 0 (0) | 0 (0) | 0.76 |
|  | Int-1 | 6 (13) | 5 (14) |  |
|  | Int-2 | 28 (61) | 20 (57) |  |
|  | High | 12 (26) | 10 (29) |  |
| Number of mutations, median (range) | | 2 | 2 | 0.92 |
| *Mutations, n (%)* | |  |  |  |
| ASXL1 | Yes | 12 (23) | 5 (14) | 0.39 |
|  | No | 40 (77) | 32 (86) |  |
| TET2 | Yes | 14 (27) | 6 (16) | 0.35 |
|  | No | 38 (73) | 31 (84) |  |
| SF3B1 | Yes | 4 (8) | 5 (14) | 0.59 |
|  | No | 48 (92) | 32 (86) |  |
| SRSF2 | Yes | 10 (19) | 9 (24) | 0.75 |
|  | No | 42 (81) | 28 (76) |  |
| IDH1/2 | Yes | 7 (13) | 6 (16) | 0.95 |
|  | No | 45 (87) | 31 (84) |  |
| Epigenetic factor mutations | Yes | 32 (62) | 18 (49) | 0.32 |
| *(TET2, DNMT3A, IDH1/2, MLL, EZH2, ASXL1 )* | No | 20 (38) | 19 (51) |  |
| Histone modulator mutations | Yes | 16 (31) | 6 (16) | 0.19 |
| *(ASXL1, EZH2)* | No | 36 (69) | 31 (84) |  |
| DNA methylation mutations | Yes | 21 (40) | 13 (35) | 0.78 |
| *(TET2, DNMT3A, IDH1/2)* | No | 31 (60) | 24 (65) |  |
| Splicing factor mutations | Yes | 18 (35) | 18 (49) | 0.27 |
| *(SF3B1, SRSF2, PRPF40B, U2AF1, U2AF35, ZRSR2)* | No | 34 (65) | 19 (51) |  |
| Cohesion factor mutations | Yes | 2 (4) | 2 (5) | 1.00 |
| *(STAG2, SMC3, PDS5B)* | No | 50 (96) | 35 (95) |  |
| Signaling factor mutations | Yes | 9 (17) | 9 (24) | 0.59 |
| *(JAK2, MPL, CBL, FLT3, NRAS, WT1, SH2B3 )* | No | 43 (83) | 28 (76) |  |
| Transcription factor mutations | Yes | 12 (23) | 8 (22) | 1.00 |
| *(RUNX1, ETV6, CEBPA, BCOR )* | No | 40 (77) | 29 (78) |  |
