# Supplementary material for: Mutations in histone modulators are associated with prolonged survival during azacitidine therapy
Source: Oncotarget. 2016 Mar 3;7(16):22103–15. doi: 10.18632/oncotarget.7899 (PMC5008347; doi:10.18632/oncotarget.7899)
Supplement: Supplementary file 3 [file oncotarget-07-22103-s003.docx]

|  |  |
| --- | --- |
|  |  |

| Table S4B: Pre-treatment variables associated with response, Kings cohort. | | |  |  |
| --- | --- | --- | --- | --- |
|  |  |  |  |  |
| Variable | | Response | No response | p-value |
| Age, median (range) | | 68 (45-82) | 67 (50-85) | 0.54 |
| Disease duration, median (range) | | 6 (1-117) | 7.5 (1-53) | 0.53 |
| Marrow blasts %, median (range) | | 11 (0-26) | 9.5 (1-19) | 0.96 |
| Cellularity %, median (range) | | 70 (20-100) | 65 (10-100) | 0.83 |
| Absolute neutrophil count, x10^9^/L, median (range) | | 1 (0.12-6.6) | 1.7 (0-8.6) | 0.86 |
| Platelets , x10^9^/L, median (range) | | 72 (20-189) | 63 (12-241) | 1.00 |
| Transfusion dependent, n (%) | Yes | 13 (57) | 15 (71) | 0.48 |
|  | No | 10 (43) | 6 (29) |  |
| Therapy-related, n (%) | Yes | 2 (9) | 3 (14) | 0.96 |
|  | No | 21 (91) | 19 (86) |  |
| IPSS cytogenetic risk group, n (%) | Favorable | 10 (43) | 2 (9) | 0.03 |
|  | Intermediate | 3 (13) | 3 (14) |  |
|  | Adverse | 10 (43) | 17 (77) |  |
| IPSS risk score, n (%) | Low | 0 (0) | 0 (0) |  |
|  | Int-1 | 5 (24) | 2 (9) |  |
|  | Int-2 | 10 (48) | 10 (45) |  |
|  | High | 6 (29) | 10 (45) |  |
| Number of mutations, median (range) | | 1 | 1 | 0.82 |
| *Mutations, n (%)* | |  |  |  |
| ASXL1 | Yes | 7 (30) | 5 (23) | 0.80 |
|  | No | 16 (70) | 17 (77) |  |
| TET2 | Yes | 4 (17) | 2 (9) | 0.70 |
|  | No | 19 (83) | 20 (91) |  |
| SF3B1 | Yes | 0 (0) | 0 (0) |  |
|  | No | 23 (100) | 22 (100) |  |
| SRSF2 | Yes | 3 (13) | 2 (9) | 1.00 |
|  | No | 20 (87) | 20 (91) |  |
| IDH1/2 | Yes | 2 (9) | 2 (9) | 1.00 |
|  | No | 21 (91) | 20 (91) |  |
| Epigenetic factor mutations | Yes | 15 (65) | 11 (50) | 0.46 |
| *(TET2, DNMT3A, IDH1/2, MLL, EZH2, ASXL1 )* | No | 8 (35) | 11 (50) |  |
| Histone modulator mutation | Yes | 9 (39) | 5 (23) | 0.39 |
| *(ASXL1, EZH2)* | No | 14 (61) | 17 (77) |  |
| DNA methylation mutations | Yes | 9 (39) | 6 (27) | 0.60 |
| *(TET2, DNMT3A, IDH1/2)* | No | 14 (61) | 16 (73) |  |
| Splicing factor mutations | Yes | 3 (13) | 4 (18) | 0.95 |
| *(SF3B1, SRSF2, PRPF40B, U2AF1, U2AF35, ZRSR2)* | No | 20 (87) | 18 (82) |  |
| Cohesion factor mutations | Yes | 0 (0) | 0 (0) |  |
| *(STAG2, SMC3, PDS5B)* | No | 23 (100) | 22 (100) |  |
| Signaling factor mutations | Yes | 1 (4) | 6 (27) | 0.09 |
| *(JAK2, MPL, CBL, FLT3, NRAS, WT1, SH2B3 )* | No | 22 (96) | 16 (73) |  |
| Transcription factor mutations | Yes | 2 (9) | 0 (0) | 0.49 |
| *(RUNX1, ETV6, CEBPA, BCOR )* | No | 21 (91) | 22 (100) |  |
